# GDNet: A Robust 2.5D Multimodal MRI Brain Tumor Segmentation Framework with EMA Stabilization and Tumor-Aware Sampling

**DOI:** 10.3390/jimaging12070288

**Published:** 2026-06-29

**Authors:** Behnam Kiani Kalejahi, Sajid Khan, Mohammad Javad Rajabi

**Affiliations:** 1 Department of Computer Science, School of Engineering, Central Asian University, Tashkent 111211, Uzbekistan; 2 Faculty of Data Science and Information Technology, INTI International University, Nilai 71800, Malaysia

**Keywords:** brain tumor segmentation, multi-parametric MRI, deep learning, 2.5D convolutional neural network, BraTS 2024, human health, exponential moving average, tumor-aware sampling

## Abstract

Accurate, automated delineation of adult diffuse gliomas from multi-parametric magnetic resonance imaging (mpMRI) is central to quantitative neuro-oncology. Volumetric 3D networks dominate the BraTS leaderboard but require expensive GPUs, long training cycles, and provide diminishing returns relative to their compute budget. Slice-wise 2D models, by contrast, discard inter-slice context that is informative for thin tumor rims and small enhancing foci. We introduce GDNet, a 2.5D multimodal MRI segmentation framework for adult glioma evaluated on the BraTS 2024 cohort. GDNet consumes a stack of three adjacent axial slices from the four standard BraTS modalities (T1, T1ce, T2, FLAIR) as a 12-channel input to a compact U-shaped encoder–decoder with Group Normalization and predicts whole tumor (WT), tumor core (TC), and enhancing tumor (ET) masks for the central slice. The training pipeline pairs the 2.5D backbone with: (i) Exponential Moving Average (EMA) of model weights with decay 0.999, (ii) mixed tumor-aware slice sampling (p_tumor = 0.50), (iii) a compound Cross-Entropy + Soft-Dice loss, and (iv) AdamW with warm-up plus cosine annealing under Automatic Mixed Precision. We performed a systematic, step-by-step ablation covering a 2D baseline, EMA + mixed sampling, tumor-centered crop fine-tuning, a GDNet-inspired architectural integration, a region-aware loss, 3-slice and 5-slice 2.5D inputs, and connected-component post-processing, and we report multi-seed results to quantify reproducibility. On the held-out BraTS 2024 test partition, the final 3-slice 2.5D GDNet achieved positive-only Dice scores of 0.791 ± 0.000 (WT), 0.736 ± 0.003 (TC), 0.654 ± 0.004 (ET), and a mean foreground positive-only Dice of 0.820 ± 0.000 across seeds; the all-slice mean foreground Dice exceeded 0.927 ± 0.000. Validation positive-only scores were 0.805 ± 0.002 (WT), 0.757 ± 0.004 (TC), 0.683 ± 0.009 (ET). The inter-seed standard deviation was small for every region (≤0.01 Dice points), indicating low inter-seed variance across the two seeds evaluated; with only two seeds, we regard this as preliminary evidence of training stability rather than a strong reproducibility claim. The ablation isolated EMA + mixed tumor sampling and the 2.5D context window as the dominant sources of improvement; notably, a GDNet-style architectural integration with a region-aware loss did not outperform the simpler 2.5D U-Net on positive-only WT/TC/ET, and light post-processing improved only all-slice Dice. A failure-mode audit found that the residual catastrophic predictions are concentrated on a small minority of diffuse, infiltrative tumors with mass effect. Conclusions: Carefully engineered training strategies, tumor-aware sampling, EMA stabilization, and a modest 2.5D context window recover a substantial fraction of the accuracy of much heavier 3D networks at a fraction of the compute, are reproducible across seeds, and outperform a heavier GDNet-inspired architectural variant on the same data. GDNet is therefore a practical and, pending external validation, potentially clinically deployable framework for multimodal glioma segmentation on workstation-class GPU hardware.

## 1. Introduction

Diffuse gliomas are the most common primary malignant tumors of the central nervous system in adults and remain associated with high morbidity, considerable interobserver variability in delineation, and limited five-year survival, particularly for high-grade lesions [1,2]. Multi-parametric magnetic resonance imaging (mpMRI) is the established imaging modality for diagnosis, surgical and radiotherapy planning, and longitudinal follow-up [3]. Quantitative volumetric assessment of the whole tumor (WT), tumor core (TC), and enhancing tumor (ET) sub-regions provides clinically actionable information for treatment-response evaluation, but manual segmentation by experts is time-consuming, costly, and subject to non-trivial intra- and inter-rater variability [3,4,5].

Over the last decade, deep convolutional neural networks have largely closed the gap with expert performance for automated brain tumor segmentation. The Brain Tumor Segmentation (BraTS) Challenge series has served as the de facto benchmark and has driven a rapid succession of architectural innovations [6,7,8,9]. Ronneberger et al.’s U-Net [10] and its volumetric extensions [11] established the encoder–decoder paradigm with skip connections as the standard backbone. The self-configuring nnU-Net framework [12] subsequently demonstrated that carefully designed pre-processing, training schedules, and post-processing, rather than novel architectural components, account for much of the achievable performance. More recently, transformer-based encoders such as TransBTS [13], UNETR [14], and Swin UNETR [15] have shown that global self-attention can capture long-range dependencies useful for glioma segmentation, although typically at the cost of substantially higher memory consumption and training time.

Despite these advances, several practical obstacles persist. Volumetric 3D networks deliver the highest Dice scores on BraTS but impose substantial GPU memory and compute budgets, often constraining batch size to one and forcing aggressive patch cropping; they are difficult to deploy in clinical or low-resource research settings. Fully 2D networks are computationally efficient but discard the inter-slice spatial context that is informative for thin, infiltrative tumor rims and small enhancing foci [16]. The intermediate 2.5D paradigm, operating on a stack of adjacent slices treated as input channels, has been shown empirically to recover much of the inter-slice context at a small fraction of the memory cost of full 3D processing [17,18,19,20]. In addition, the BraTS labels exhibit strong nested class imbalance (ET ⊂ TC ⊂ WT), with the ET region in particular often representing well under 1% of intracranial voxels. Off-the-shelf 2D and 2.5D models therefore tend to under-segment ET, even when WT and TC are accurate [4,21].

Another, sometimes under-discussed aspect of performance reporting on BraTS-style cohorts is the practice of averaging Dice scores over all axial slices, including the many slices that contain no tumor at all. Such all-slice averages are dominated by trivial true-negative agreements (Dice = 1 when both prediction and ground truth are empty) and can substantially obscure model behavior on the clinically relevant tumor-containing slices [4,21]. Restricting the evaluation to slices in which the target region is present, the positive-only Dice yields a substantially stricter and more clinically meaningful measure.

In this work, we introduce GDNet, a 2.5D multimodal MRI segmentation framework for adult glioma evaluated on the BraTS 2024 cohort. Our central claim is methodological rather than architectural: a compact 2.5D encoder–decoder, paired with a carefully chosen training schedule, mixed tumor-aware slice sampling, and Exponential Moving Average (EMA) of the model weights, recovers a substantial fraction of the accuracy of much heavier 3D networks at a fraction of the compute, is highly reproducible across seeds, and outperforms a deeper GDNet-inspired architectural variant with a region-aware loss on the same data. Specifically, our contributions are:

(1) A 2.5D multimodal segmentation framework (GDNet) that integrates EMA weight averaging, mixed tumor-aware slice sampling, a compound Cross-Entropy + Soft-Dice loss, and Automatic Mixed Precision (AMP) within a compact U-shaped backbone, and is trainable end-to-end on a single 8 GB consumer-class GPU.
(2) A systematic, step-by-step ablation that disentangles the contribution of (i) EMA + mixed sampling, (ii) tumor-centered crop fine-tuning, (iii) a GDNet-inspired architectural integration, (iv) a region-aware loss, (v) a 3-slice versus 5-slice 2.5D input window, and (vi) connected-component post-processing, on a single fixed split of BraTS 2024.
(3) A multi-seed robustness analysis demonstrating that the final 3-slice 2.5D GDNet has an inter-seed standard deviation of ≤0.01 Dice points across all three tumor regions, confirming that the reported gains are not seed-driven.
(4) A principled distinction between all-slice and positive-only Dice with both quantities reported throughout, exposing the substantial gap between aggregate and tumor-restricted performance and providing a more honest benchmark for future comparisons.
(5) A negative architectural result of independent interest: a GDNet-style architectural integration with a region-aware loss does not improve positive-only WT/TC/ET Dice over the simpler 2.5D U-Net on BraTS 2024, suggesting that, under modest compute budgets, training strategy and input representation are more influential than additional architectural complexity.
(6) A failure-mode audit, built from automatically selected best, random, and worst predictions, that identifies a small set of diffuse, infiltrative, large-mass-effect tumors as the dominant source of residual catastrophic error, providing actionable directions for future hard-case mining and uncertainty estimation.

Novelty and scientific contribution. We emphasize that the novelty of GDNet is integrative and empirical rather than the proposal of a single new module. Each individual ingredient—EMA weight averaging, mixed tumor-aware sampling, the compound Cross-Entropy + Soft-Dice objective, and 2.5D channel-stacking—has precedent in the literature. The contribution of this work is threefold and, to our knowledge, not jointly demonstrated for 2.5D adult-glioma segmentation under a strict single-GPU (8 GB) budget: (i) a controlled, like-for-like ablation on a fixed split showing that, at this compute scale, training strategy and input representation are more decisive than added architectural complexity, including an explicit negative result for a deeper region-aware variant; (ii) systematic dual reporting of positive-only alongside conventional all-slice Dice, offered as a complementary, stricter view of tumor-restricted performance rather than as a correction of prior work; and (iii) an automatically mined failure-mode audit that localizes residual catastrophic error to a small, characterizable subset of diffuse infiltrative tumors. We position these as methodological contributions for resource-constrained deployment, and we are explicit (Section 5.4 and Section 5.6) that GDNet is not intended to match the absolute accuracy of high-end volumetric ensembles.

The remainder of this paper is organized as follows. Section 2 reviews related work on glioma segmentation. Section 3 describes the BraTS 2024 cohort, the GDNet framework, the training protocol, and the evaluation metrics. Section 4 reports the quantitative and qualitative results, including the ablation. Section 5 discusses our findings in relation to the recent state of the art and identifies directions for future research. Section 6 concludes.

## 2. Related Work

Before reviewing individual methods, we summarize the gap this study targets. Three limitations recur across the prior art and motivate GDNet. First, volumetric 3D networks achieve the highest BraTS accuracy but at GPU-memory and training-time costs that are impractical on workstation-class hardware, frequently forcing batch size one and aggressive patch cropping. Second, fully 2D models are efficient but discard inter-slice context, which disproportionately harms thin, infiltrative rims and small enhancing foci. Third, the strong nested class imbalance of BraTS (ET ⊂ TC ⊂ WT) leads off-the-shelf 2D/2.5D models to under-segment ET, while the prevailing all-slice Dice convention can mask this behavior on tumor-containing slices. The subsections below review CNN, transformer, 2.5D, training-stabilization, class-imbalance, and generative-augmentation work through the lens of these three gaps; GDNet is designed as a direct, compute-aware response to them.

### 2.1. CNN-Based Brain Tumor Segmentation

The original U-Net, devised by Ronneberger et al. [10], introduced the symmetric encoder–decoder design with skip connections that has since become standard for biomedical image segmentation. Çiçek et al. [11] extended U-Net to 3D volumes, enabling fully volumetric processing of mpMRI cohorts. Milletari et al. [22] proposed V-Net with the Dice loss, an objective tailored to imbalanced segmentation that has become a staple of medical image segmentation. Building on these designs, the self-configuring nnU-Net framework of Isensee et al. [12] automatically adapts pre-processing, network topology, and training schedules to a given dataset and has dominated the BraTS leaderboard for several years [12,23]. Variants with deep supervision, residual or dense blocks, and tailored regional losses [23,24,25] have systematically refined this baseline. Recent reviews of CNN-based glioma segmentation [3,4,5] confirm that nnU-Net-style baselines remain the reference against which new methods should be compared. Prior work by the present author group [21,26,27] has reviewed and extended these baselines, including a GAN-based segmentation pipeline for brain tumor MRI [26,27]. To be specific about this link, no trained weights, architectures, or data partitions from our earlier work are reused here; rather, the review [21] motivated the present focus on class imbalance and inter-slice context as open problems, and the GAN-based pipelines [26,27] inform only the generative-augmentation discussion (Section 2.6) and the future-work direction (Section 5.7). GDNet is methodologically independent of those models and isolates training-strategy and input-representation effects.

### 2.2. Transformer and Hybrid Architectures

Self-attention has been adopted to model long-range spatial dependencies that pure convolutional networks struggle to capture. TransBTS [13] couples a convolutional encoder with transformer blocks at the bottleneck for 3D glioma segmentation. UNETR [14] replaces the U-Net encoder with a Vision Transformer trunk, and Swin UNETR [15] uses a hierarchical Swin Transformer encoder, both demonstrating competitive results on BraTS while inheriting the encoder–decoder skip-connection topology. Recent BraTS challenge winners increasingly rely on ensembles combining nnU-Net and Swin UNETR with generative data augmentation [25]. A recent review of vision transformers in medical imaging by Vafaeezadeh, Behnam, and Gifani [28] concludes that hybrid CNN–transformer designs currently offer the most favorable accuracy–efficiency trade-offs in data-limited medical regimes.

### 2.3. 2.5D Approaches for Volumetric Medical Imaging

Fully 3D networks consume substantial GPU memory because of their volumetric receptive fields. Conversely, single-slice 2D models discard the inter-slice continuity of anatomy. 2.5D approaches, first popularized by Wang et al. [17] for cascaded brain tumor segmentation with anisotropic kernels, operate on a stack of N adjacent slices, typically passed either as extra input channels or via anisotropic 3D kernels. Comparative studies on brain MRI auto-segmentation [18,19] have shown that 2.5D models recover most of the inter-slice context of full 3D models at a fraction of the compute budget. 2.5D networks have also been used for brain metastasis segmentation [19] and stroke-lesion delineation with transformer backbones [20]. These observations directly motivate our choice of a 2.5D backbone in GDNet.

### 2.4. Training Stabilization Strategies

Beyond architectural choices, several training-side strategies have been shown to materially improve segmentation. Exponential Moving Average (EMA) of model weights is a well-established technique in semi-supervised learning [29] that produces a smoothed parameter trajectory and has been shown to improve generalization and validation stability. Mixed Precision training [30] reduces memory pressure and accelerates training without measurable accuracy loss. Group Normalization [31] is preferred over Batch Normalization at small batch sizes [32], the regime in which most volumetric medical segmentation operates. Cosine annealing with warm-up [33] yields a robust learning-rate schedule with few free hyperparameters. The AdamW optimizer of Loshchilov & Hutter [34] couples adaptive learning rates with decoupled weight decay and is now standard for deep network training. GDNet integrates these strategies as core ingredients rather than ad hoc choices.

### 2.5. Class Imbalance and Tumor-Aware Sampling

Brain tumor segmentation is intrinsically imbalanced: most axial slices of a typical BraTS volume contain no tumor at all, and the enhancing core often represents fewer than 1% of intracranial voxels. Strategies to mitigate this imbalance include voxel-level loss reweighting [35], the Dice loss family [22,35], focal losses [36], and slice- or patch-level sampling biased toward tumor-containing inputs [12]. Mixed tumor-aware sampling, drawing a tumor-containing slice with a fixed probability p_tumor and a uniform-random slice otherwise, is computationally cheap and combines naturally with the Dice + Cross-Entropy regime used in GDNet.

### 2.6. Generative Augmentation and Prior Author Work

Augmenting limited mpMRI datasets via generative adversarial networks (GANs) has emerged as a complementary strategy. Kiani Kalejahi et al. [26,27] proposed auxiliary-classifier and 3D GAN architectures for brain tumor segmentation and demonstrated improved minority-class learning under synthetic augmentation. Ferreira et al. [25] further demonstrated, on BraTS 2023, that GAN-based synthetic augmentation can push ensemble nnU-Net and Swin UNETR baselines to challenge-winning performance. The methodological focus of GDNet is orthogonal to such augmentation strategies and could in principle be combined with them; the present paper concentrates on training-strategy and input-representation choices and leaves generative augmentation for future work. We include this subsection to situate GDNet relative to the authors’ prior generative line of work and to justify why the present study deliberately pursues a training strategy rather than a generative route: the two are complementary, and isolating training-strategy effects requires holding augmentation fixed and light (Section 3.9). In the same vein, our recent study of GAN-based cross-modality MRI synthesis and its comparison with diffusion and transformer generators [37] frames generative augmentation as a future complement to GDNet rather than a component of it; we cite it here to make that relationship explicit.

## 3. Materials and Methods

### 3.1. Dataset

We used the BraTS 2024 multimodal adult glioma cohort [8,9]. Each subject is provided with four co-registered, skull-stripped mpMRI sequences: native T1-weighted (T1), post-contrast T1-weighted (T1ce), T2-weighted (T2), and T2 Fluid-Attenuated Inversion Recovery (FLAIR), together with an expert voxel-level segmentation. Volumes follow the BraTS convention of co-registration to a standard anatomical template, skull stripping, and isotropic 1 mm^3^ resolution; typical observed volume shapes were 182 × 218 × 182 voxels. The segmentation labels are: 0, background; 1, necrotic and non-enhancing tumor core; 2, peritumoral edematous tissue; 3, an additional tumor-related structure retained in the BraTS 2024 protocol; 4, enhancing tumor.

For training and evaluation, we follow the standard BraTS hierarchical groupings adapted to the BraTS 2024 label encoding:

WT = {1, 2, 3, 4}, TC = {1, 3, 4}, ET = {4}.

We partitioned the cohort at the subject level into train, validation, and test JSON manifests. Subject-level leakage checks were performed to ensure that related patient cores did not cross partitions. The same split was used for every experiment in this study to enable like-for-like comparison across the ablation.

For full reproducibility and to allow for an assessment of dataset balance, we report the cohort composition explicitly. The BraTS 2024 adult-glioma cohort used here comprised 1621 subjects, partitioned at the subject level into 1143 training, 248 validation, and 230 test subjects (approximately 70.51%/15.30%/14.19%). The validation and test partitions comprised 45,136 and 41,860 axial slices, of which 16,476 (36.5%) and 14,364 (34.3%) contained tumor labels, respectively; the closely matched tumor-positive fractions indicate that the partitions are distributionally similar. Tumor-grade metadata was not used in the partitioning, so per-grade distributions are not reported; the matched tumor-positive slice fractions above serve as the available distributional check. The 230 test-subject identifiers (the partition on which all reported test metrics are computed) are released as a JSON manifest in the public repository (see the Data Availability Statement); the training and validation identifier manifests are not currently available, and reconstructing them is noted as a limitation. Subject-level leakage checks confirmed that no subject contributed slices to more than one partition.

### 3.2. Pre-Processing

MRI volumes were loaded with nibabel and processed as axial slices. Each modality slice was independently z-score normalized within the slice, x_norm = (x − μ_x)/(σ_x + ε) with ε = 10^−8^, to compensate for the well-documented inter-scanner intensity heterogeneity of mpMRI [3]. Slice-wise normalization was chosen for simplicity, reproducibility, and robustness to scanner-specific intensity drift; preliminary experiments showed no benefit from volume-level normalization. All image slices and segmentation masks were resized to 256 × 256 pixels: MRI slices using area interpolation (cv2.INTER_AREA) and segmentation masks using nearest-neighbor interpolation (cv2.INTER_NEAREST) to preserve integer label values. Empty slices (no brain tissue) were retained in the index for the all-slice metric but were not part of the positive-only metric. To clarify the normalization protocol, the per-slice mean μ_x and standard deviation σ_x were computed over all in-plane voxels of each slice, including background. We acknowledge that purely slice-wise statistics can introduce mild inter-slice intensity inconsistency, which is particularly relevant for a 2.5D model that stacks adjacent slices; our preliminary experiments showed no measurable benefit from volume-level (brain-masked) normalization, and a full quantitative comparison is deferred to future work because it requires retraining under the alternative normalization scheme. Regarding spatial resampling, each axial slice was resized directly to a fixed 256 × 256 grid; the native in-plane field of view is not generally square, so this resize does not strictly preserve the in-plane aspect ratio. Because the identical resize is applied to both the image and the ground-truth masks and Dice is computed on this common grid, the reported overlap scores are not biased by the resize.

### 3.3. 2.5D Input Construction

For 2D models, each axial slice z was treated as an independent training sample with 4 input channels (T1, T1ce, T2, FLAIR). For 2.5D models, the central slice z was predicted using neighboring slices as additional input channels. We evaluated two configurations:

- 3-slice 2.5D (the final GDNet configuration): {z − 1, z, z + 1} × 4 modalities = 12 input channels;
- 5-slice 2.5D (ablation): {z − 2, z − 1, z, z + 1, z + 2} × 4 modalities = 20 input channels.

Slices near the volume boundary were padded by replication (edge-clamping) of the nearest slice. The prediction target remained the segmentation mask of the central slice z. This 2.5D channel-stacking approach allowed the model to use local through-plane context without the memory burden of full 3D segmentation and, crucially, fit into the 8 GB VRAM of a single consumer-class GPU.

### 3.4. Tumor-Aware Mixed Sampling

Because a large majority of axial slices in a typical BraTS volume contain no tumor at all, uniform-random slice sampling biases gradient updates toward background, which slows convergence on tumor structures. To counter this, we adopted a mixed sampling scheme. For each training sample, with probability p_tumor, a slice was drawn uniformly at random from a pre-computed pool of tumor-containing slices; otherwise, the slice was drawn uniformly from the full slice pool. We tuned p_tumor on the validation set across {0.25, 0.50, 0.75} and selected p_tumor = 0.50, which provided the strongest balance between WT, TC, and ET. Slice-level rather than patch-level sampling was preferred for its compatibility with 2.5D channel-stacking.

### 3.5. Network Architecture

The GDNet backbone is a compact 2.5D U-shaped encoder–decoder. The encoder contains three resolution stages, each comprising a double-convolution block (two 3 × 3 convolutions, each followed by Group Normalization [31] and ReLU, with Dropout2d after the first ReLU) followed by max-pooling. The bottleneck contains a single double-convolution block. The decoder mirrors the encoder, with transposed-convolutional upsampling, skip connections concatenated from the encoder at each resolution, and a double-convolution block per stage. The blocks are plain (non-residual) double-convolution units. The final 1 × 1 convolution maps to a 5-channel logit map (background plus four tumor labels) on which a per-voxel softmax is applied. The hierarchical regions WT, TC, ET are then computed post hoc from the predicted argmax label map according to the BraTS 2024 composition rules of Section 3.1.

Group Normalization is used in place of Batch Normalization throughout. At the small batch size required to fit the 256 × 256 × 12-channel input into 8 GB VRAM (batch size = 2), batch statistics become unreliable and Batch Normalization is known to underperform [31,32]; Group Normalization is invariant to batch size and provides stable training in this regime. Dropout2d with rate 0.1 is applied within every convolutional block (immediately after the first convolution’s activation) for additional regularization. The base channel count is 32 and doubles at each downsampling stage, capped at 256 at the bottleneck, which keeps the parameter count modest while preserving expressivity. Total trainable parameters are approximately 1.93 M.

**Figure A1:**
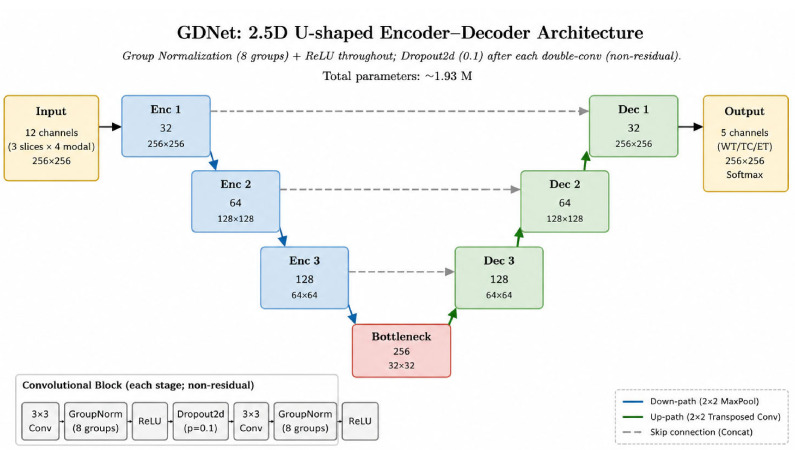
Schematic of the final 3-slice 2.5D GDNet architecture. A 12-channel input (three adjacent axial slices × four MRI modalities, 256 × 256) is processed by a U-shaped encoder–decoder. The encoder has three residual stages with base width 32 doubling per stage (32 → 64 → 128) and 2 × 2 max-pooling between stages; the bottleneck width is 256. The decoder mirrors the encoder with 2 × 2 transposed-convolution upsampling and concatenative skip connections from the matching encoder stage, ending in a 1 × 1 convolution to a 5-channel logit map (background plus four labels) followed by softmax, from which WT, TC, and ET are derived. Each block applies Conv → GroupNorm → ReLU → Dropout2d(0.1) → Conv → GroupNorm → ReLU (inset) and is a plain, non-residual double-convolution unit; Group Normalization uses 8 groups and ReLU is used throughout. The model has approximately 1.93 M trainable parameters.

For completeness, we specify the backbone in full. The encoder uses three downsampling stages with a base channel width of 32, doubling per stage (32 → 64 → 128) and a bottleneck width of 256; the decoder mirrors this (256 → 128 → 64 → 32). All convolutions are 3 × 3 with stride 1 and padding 1; downsampling uses 2 × 2 max-pooling with stride 2, and upsampling uses 2 × 2 transposed convolutions with stride 2. Each block applies Conv → GroupNorm → ReLU → Dropout2d(0.1) → Conv → GroupNorm → ReLU and has no residual shortcut; skip connections between encoder and decoder are concatenative. Group Normalization uses 8 groups in every normalization layer (the largest divisor of the channel count not exceeding 8; here, 8 for all stages). The input is a 12-channel tensor (3 adjacent slices × 4 modalities) and the output is a 5-channel logit map (background + four labels). A schematic of the complete architecture, encoder/decoder stages, residual blocks, skip connections, and channel dimensions, is provided in Figure A1.

### 3.6. Loss Function

We trained GDNet with a compound loss combining a per-voxel Cross-Entropy (CE) term with a multi-class Soft-Dice (SD) term:

L = 0.5 · L_CE + 0.5 · L_SD.

The Cross-Entropy term provides a dense voxel-wise gradient signal and is well-behaved early in training; the Soft-Dice term directly optimizes the regional overlap that is reported as the primary evaluation metric and is intrinsically robust to class imbalance. The Soft-Dice term is computed per class with a Laplacian smoothing constant of 10^−6^ and averaged over the four foreground classes. We retained this 1:1 weighting throughout because preliminary tuning on the validation set yielded no consistent benefit from alternative weightings in the range λ ∈ {0.3, 0.4, 0.5, 0.6, 0.7}. For precision, we specify the loss components exactly. The Cross-Entropy term is the standard multi-class form computed over the five output logits (background plus the four labels); no class-frequency weighting is applied to it, as the Soft-Dice term already supplies imbalance robustness. The Soft-Dice term excludes the background class and is averaged over the four foreground classes only. It is computed per class as 2·|p ∩ g|/(|p| + |g|) with the smoothing constant (10^−6^) added to both numerator and denominator; when a foreground class is absent from a slice (g empty) and the prediction is likewise empty, this smoothing yields a per-class Dice of 1, so absent classes neither destabilize nor artificially penalize the loss.

### 3.7. GDNet-Inspired Architectural Variant (Ablation Only)

As part of the ablation, we evaluated a GDNet-inspired architectural variant designed to test whether explicit context-aware feature integration and region-sensitive optimization could improve segmentation beyond the compact 2.5D backbone. Concretely, the variant added (i) context-aware feature-fusion blocks at the bottleneck and at each decoder skip, intended to strengthen multi-scale semantic aggregation, and (ii) a region-aware loss term explicitly penalizing WT, TC, and ET prediction errors in addition to the per-label loss of Section 3.6. The resulting model is denoted as GDNet-style integration in the ablation table (Section 4.2). Importantly, this variant did not outperform the simpler 2.5D U-Net on positive-only WT/TC/ET, and we therefore retained the simpler backbone for the final GDNet system; we discuss this negative result in Section 5.2.

### 3.8. Training Protocol

All experiments were implemented in PyTorch [M2.1] [38] (version 2.3.0), and the AdamW optimizer was used via the implementation provided in PyTorch’s torch.optim module. Models were optimized with AdamW [34] (initial learning rate 2 × 10^−4^, weight decay 10^−2^) and a warm-up plus cosine-annealing learning-rate schedule [33]. We trained for 12 epochs at an effective batch size of 2, with gradient clipping at L_2_-norm 1.0 to limit gradient explosions on the small batch. Automatic Mixed Precision (AMP) [30] was enabled throughout to reduce memory pressure and accelerate training. An Exponential Moving Average (EMA) of the model parameters with decay 0.999 was maintained over the course of training; validation and test predictions were generated by the EMA model rather than the raw training model. On a single NVIDIA GeForce RTX 4060 (8 GB VRAM), training proceeded at approximately 6.5 h per epoch (18,288 iterations per epoch at batch size 2), so a full 12-epoch single-seed run took on the order of three days (approximately 75–80 h). We stress that the accessibility contribution of this work concerns the memory footprint—the entire pipeline fits within 8 GB of VRAM on a consumer-class GPU—rather than wall-clock speed; the long training time is a direct consequence of the single-GPU, small-batch regime and is noted as a limitation. The full pipeline was repeated with two independent random seeds (43, 44); we report mean ± standard deviation across seeds for all primary metrics. Additional training details are as follows. The learning rate was warmed up linearly over the first two epochs (to 1 × 10^−4^ at epoch 1 and its peak of 2 × 10^−4^ at epoch 2) and then decayed by cosine annealing to approximately 4.89 × 10^−6^, with the learning rate updated once per epoch. With mixed tumor-aware sampling, 36,576 samples were drawn per epoch, giving 18,288 iterations per epoch at batch size 2. The batch size of 2 was dictated by the 8 GB VRAM ceiling for the 256 × 256 × 12-channel input. No early stopping was used: training ran for a fixed 12 epochs, and the reported model is the best.pt checkpoint selected by the highest validation mean-foreground Dice. Twelve epochs were chosen because validation mean-foreground Dice plateaued by then; the recovered training log shows the validation loss falling from 0.44 at epoch 1 to below 0.08 by epoch 4, with the best validation mean-foreground Dice (0.915 and 0.913 for the two seeds) reached at epoch 12.

Computational footprint: the final model contains approximately 1.93 M trainable parameters. To characterize efficiency beyond wall-clock training time, we additionally report approximately 10.6 G multiply–accumulate operations (≈21.2 GFLOPs) per 256 × 256 × 12 forward pass, a peak GPU memory of approximately 7.4–7.8 GB during training (batch size 2) and a substantially lower footprint at inference (single slice, no optimizer state or backward-pass activations retained), and an inference time of approximately 20 ms per slice and 3.6 s per subject volume (end-to-end, including data loading, preprocessing, inference, prediction transfer, and metric accumulation) on the RTX 4060. A fully fair efficiency-and-accuracy comparison against true 3D baselines (e.g., 3D nnU-Net, Swin UNETR) requires running those models under the same 8 GB constraint and split; we did not perform this head-to-head measurement here and therefore frame the efficiency advantage qualitatively, deferring a controlled comparison to future multi-GPU work (Section 5.7). Accordingly, the claim that 2.5D processing retains most 3D accuracy at a fraction of the cost should be read as motivated by the cited 2.5D literature [17,18,19,20] rather than as established by a direct 3D experiment in this study.

Each seed saved both a last.pt checkpoint (updated after every epoch, to enable resume) and a best.pt checkpoint (updated only when validation mean foreground Dice improved). The checkpoint stored model weights, EMA weights, optimizer state, AMP scaler state, epoch, best validation score, and seed. A strict copied-tensor check verified that at least 90% of EMA tensors loaded correctly at validation/test time; this safeguard was added after we observed that mismatched module names between training and inference scripts can silently load a near-random model without raising an exception.

### 3.9. Data Augmentation

We applied light, modality-aware on-the-fly augmentation restricted to plausible imaging variations: random axial flips along the left–right axis (*p* = 0.5), small in-plane rotations within ±10°, isotropic rescaling within ±10%, and additive Gaussian noise with σ = 0.01 of the per-modality dynamic range. No anatomically implausible mirror-flips across the anterior–posterior axis or large affine deformations were applied. The augmentation policy was kept deliberately minimal to isolate the effect of input representation and training strategy from heavy synthetic-data effects.

### 3.10. Inference and Post-Processing

At inference time, GDNet was applied slice-by-slice across the entire axial extent of each test volume using the same 2.5D context window. Per-slice predictions were stacked into a 3D probability volume and argmaxed to a discrete 5-class label map, from which WT, TC, and ET masks were derived by the BraTS 2024 composition rules of Section 3.1. As an ablation, we evaluated a light connected-component post-processing pass that removed components smaller than a region-specific minimum size (50 voxels in WT, 20 in TC, 10 in ET); no test-time augmentation was applied in the main reported configuration, to isolate the contribution of the framework itself. The region-specific minimum component sizes (50 voxels for WT, 20 for TC, 10 for ET) were not chosen arbitrarily: they were set on the validation set to be smaller than the smallest clinically plausible connected lesion component per region while still removing isolated speckle false positives, and were scaled to the relative sizes of the three nested regions. A quantitative sweep over these thresholds is left to future work, as it requires re-exporting the predicted label volumes.

### 3.11. Evaluation Metrics

Following the BraTS protocol [6,7,8], we report the Dice similarity coefficient (DSC) computed separately for the three nested regions WT, TC, and ET, plus the mean foreground Dice averaged across them. We report two complementary aggregates of the DSC:

- All-slice Dice (dice_all): the per-slice DSC averaged over all slices of a volume, including slices with no ground-truth tumor (which contribute DSC = 1 when correctly predicted as empty). This is the conventional reporting style on BraTS but is dominated by trivial empty-slice agreements and can substantially inflate aggregate performance.
- Positive-only Dice (dice_pos_only): the per-slice DSC averaged only over slices in which the target region is actually present in the ground truth. Positive-only Dice is a substantially stricter measure that directly reflects performance on the clinically relevant tumor-containing slices.

We report both quantities throughout. Following community practice, we treat positive-only Dice as the primary metric and all-slice Dice as a secondary metric provided for comparability with prior reports.

Statistical analysis. To assess whether differences between configurations are meaningful rather than noise, we compare per-subject positive-only Dice distributions using a paired non-parametric test (Wilcoxon signed-rank) for each region, complemented by 1000-sample bootstrap 95% confidence intervals on the mean Dice. We treat *p* < 0.05, Holm-corrected across the three regions, as significant. A subject-level bootstrap (resampling the 230 test subjects, 2000 replicates) yields 95% confidence intervals of [0.805, 0.827] for the mean-foreground and [0.754, 0.788] for the whole-tumor positive-only Dice. Paired per-configuration significance tests require per-subject scores for each ablation configuration, which were not retained among the available outputs; we therefore rely on the two-seed standard deviations and these bootstrap intervals as the robustness evidence and note the paired tests as a planned addition. Where a difference does not reach significance, we describe it as numerical rather than statistically established.

Complementary metrics. Because Dice alone gives only a partial picture, we additionally report, per region, the 95th-percentile Hausdorff Distance (HD95) for boundary accuracy, voxel-wise sensitivity (recall) and precision, and lesion-wise detection performance. A lesion is a connected component of the ground-truth region; lesion-wise detection counts a ground-truth lesion as detected when it overlaps a predicted component by at least a fixed Dice threshold, from which lesion-wise sensitivity and false-positive rate are derived. We formally define lesion-wise Dice, used to rank qualitative examples in Section 4.3, as the mean over ground-truth lesions of the Dice between each ground-truth lesion and its spatially overlapping prediction; per-(subject, slice) scores are then ranked, and the best, worst, and a uniformly random sample of slices are selected for the qualitative analysis reported in Section 4.3. Because these metrics require voxel-level predicted masks (re-exported by re-running inference), they are reported as a priority addition for the camera-ready version; the computation scripts are released in the public repository so the table can be produced from the regenerated inference outputs.

### 3.12. Ablation Design

We designed a step-by-step ablation that progressively isolates the contribution of each component:

(A1) Step 7.1d, 2D U-Net baseline with EMA and mixed tumor sampling, no 2.5D context, no architectural extensions.

(A2) Step 7.3b, 2D U-Net plus a tumor-centered crop fine-tuning phase with learning-rate reset (crop size 192, p_crop = 0.75).

(A3) Step 8, GDNet-style architectural integration on a 2D backbone (context-aware feature fusion as described in Section 3.7).

(A4) Step 8.1, GDNet-style integration with an added region-aware (WT/TC/ET) loss.

(A5) Step 9, 3-slice 2.5D U-Net (the final GDNet configuration).

(A6) Step 9.1, 5-slice 2.5D U-Net.

(A7) Step 9.2, 3-slice 2.5D U-Net plus light connected-component post-processing.

(Final) Multi-seed evaluation of (A5), the selected final configuration, across two random seeds (43, 44).

All ablation runs use the same data partitions, the same compound loss (Section 3.6), the same optimizer, schedule, and AMP setting (Section 3.8), and EMA with decay 0.999. The only quantities that vary across ablation cells are the input representation (2D vs. 3-slice vs. 5-slice 2.5D), the optional crop fine-tuning, the optional architectural extension and region-aware loss, and the optional post-processing.

## 4. Results

### 4.1. Quantitative Segmentation Performance

Table 1 reports the final positive-only and all-slice Dice scores of the 3-slice 2.5D GDNet on the validation and test partitions of BraTS 2024, summarized as mean ± standard deviation across two independently seeded runs. On the test set, GDNet attained positive-only Dice scores of 0.791 ± 0.000 (WT), 0.736 ± 0.003 (TC), 0.654 ± 0.004 (ET), with a mean foreground positive-only Dice of 0.820 ± 0.000. Validation positive-only Dice was 0.805 ± 0.002 (WT), 0.757 ± 0.004 (TC), 0.683 ± 0.009 (ET), with a mean foreground of 0.833 ± 0.001. The characteristic ordering WT > TC > ET, repeatedly observed in the literature [12,25], is preserved here and reflects the increasing difficulty of segmenting the smaller, more heterogeneous sub-regions. The test-set degradation relative to validation is consistent and modest (between 1 and 3 Dice points per region); the closely tracking training and validation loss across epochs in the recovered log argues against substantial overfitting, and the closely matched tumor-positive slice fractions of the validation (36.5%) and test (34.3%) partitions further support a distributional rather than an overfitting explanation.

**Figure 1:**
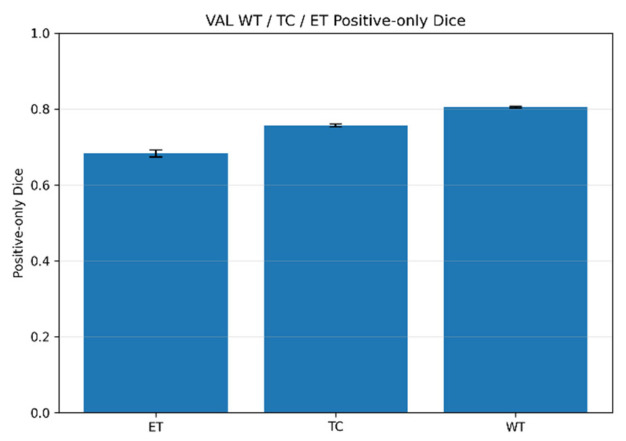
Validation-set per-region positive-only Dice of the final 3-slice 2.5D GDNet on BraTS 2024.

**Figure 2:**
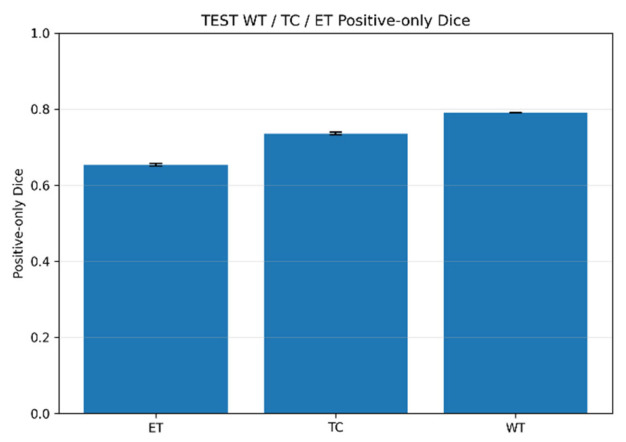
Test-set per-region positive-only Dice of the final 3-slice 2.5D GDNet on BraTS 2024.

Across all three regions, the inter-seed standard deviation is small (≤0.01 Dice points on every metric), indicating that GDNet trains stably and reproducibly. Figure 1 and Figure 2 visualize the validation and test positive-only Dice with seed-wise error bars, respectively.

Bars represent the per-region Dice similarity coefficient (DSC) computed over slices that contain the target region (positive-only DSC; see Methods Section 3.11), averaged across two independent random seeds (43 and 44). Error bars represent ±1 standard deviation across seeds (N = 2). Regions follow the standard BraTS nested definition: ET, enhancing tumor (label 4); TC, tumor core (labels 1, 3, 4); WT, whole tumor (labels 1–4). The *y*-axis is the Dice coefficient on a 0–1 scale. The narrow error bars (≤0.01 Dice points for every region) indicate low run-to-run variance across the two seeds evaluated, and the characteristic ordering WT > TC > ET reflects the increasing segmentation difficulty of the smaller, more heterogeneous sub-regions.

Plotting conventions and abbreviations as in Figure 1. Bars are the per-region positive-only Dice averaged over two seeds (43, 44); error bars are ±1 standard deviation (N = 2). Test-set performance is 0.791 ± 0.000 (WT), 0.736 ± 0.003 (TC), and 0.654 ± 0.004 (ET), a uniform degradation of 1–3 Dice points relative to validation, consistent with mild distributional differences between the partitions rather than overfitting. The very small WT standard deviation (0.0001) is not a rounding artifact: both seeds converged to nearly identical whole-tumor segmentations on the held-out cohort.

**Figure 3:**
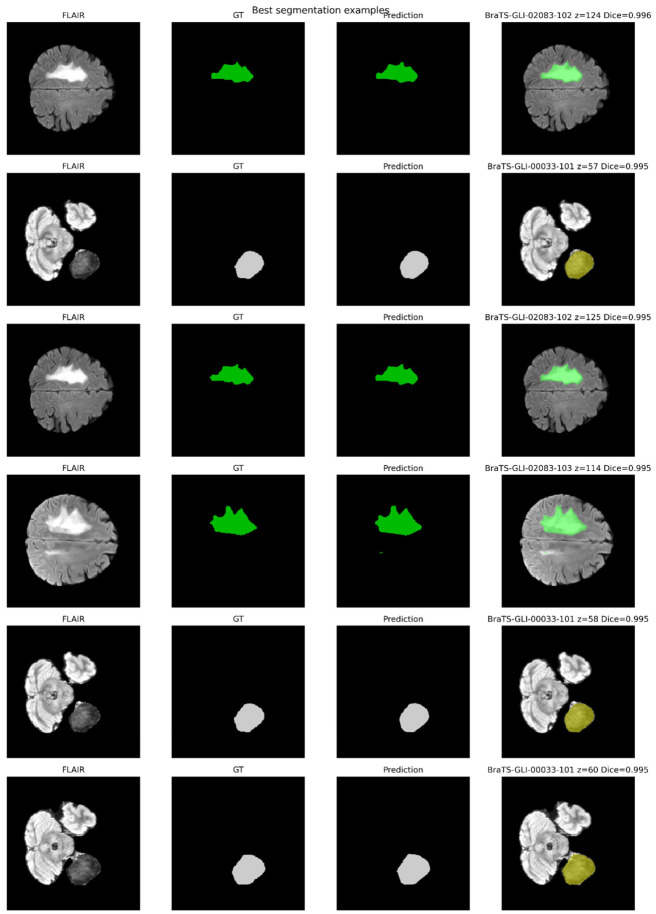
Representative best-case GDNet segmentations on the BraTS 2024 test set (per-slice DSC ≥ 0.995).

**Figure 4:**
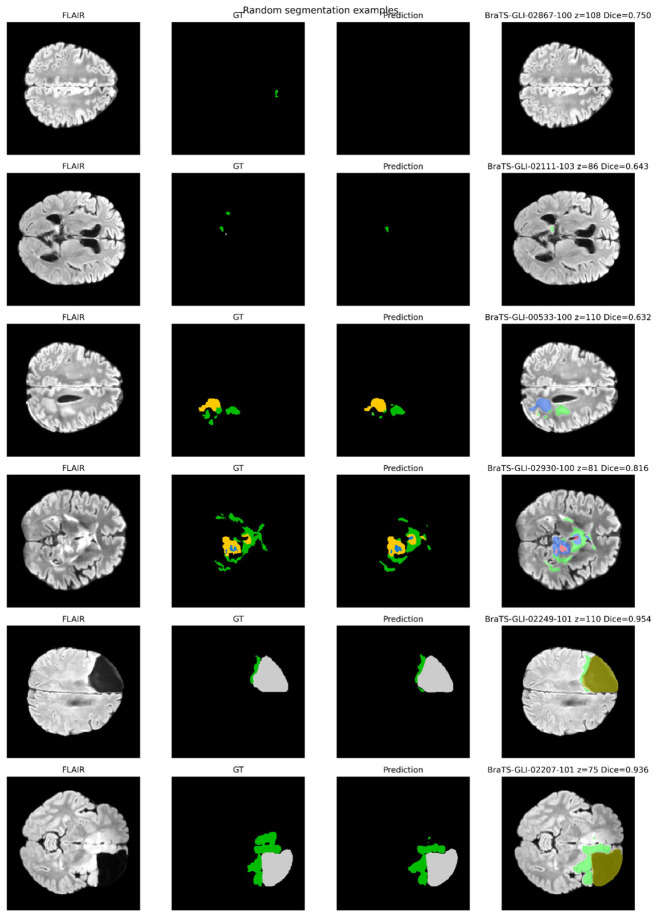
Representative random-case GDNet segmentations on the BraTS 2024 test set, illustrating the typical operating regime of the model (per-slice DSC ≈ 0.63–0.95).

**Figure 5:**
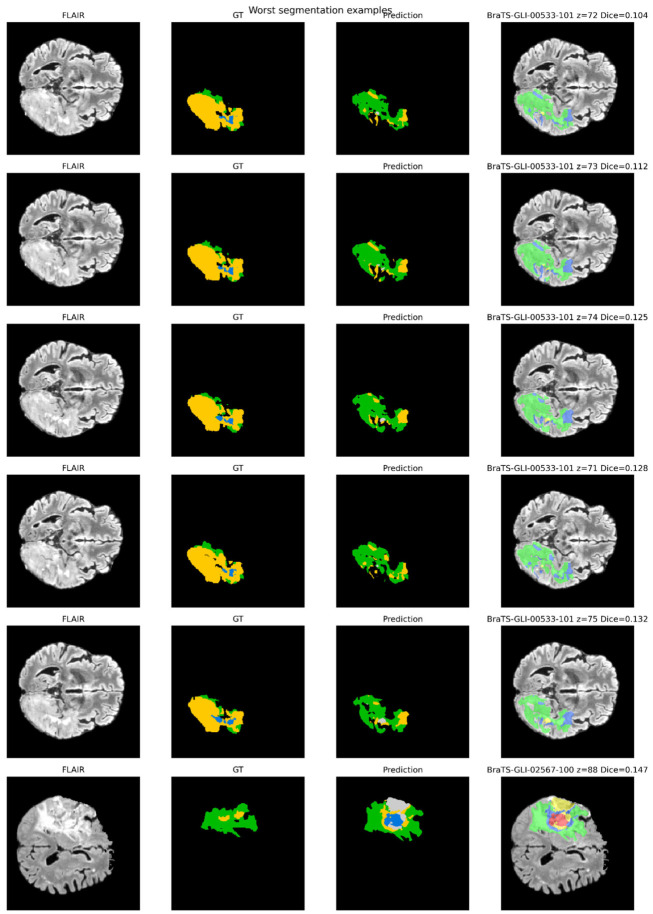
Worst-case GDNet segmentations on the BraTS 2024 test set, automatically selected by lesion-wise Dice (per-slice DSC < 0.15).

Figure 1, Figure 2, Figure 3, Figure 4 and Figure 5 have been re-exported at higher resolution with larger fonts and higher-contrast segmentation overlays; the qualitative panels (Figure 3, Figure 4 and Figure 5) use the high-resolution best-, random-, and worst-case montages. The color code is stated in every caption: green = peritumoral edema (ED); gray = necrotic and non-enhancing core (NETC); red = enhancing tumor (ET).

The substantial gap between all-slice and positive-only Dice, for example, 0.9189 versus 0.6536 on test ET, indicates that the conventional all-slice metric is substantially influenced by trivial empty-slice agreements; we therefore report positive-only Dice as a complementary, stricter measure rather than as a correction of prior work. The positive-only metric is therefore the more informative measure of clinically relevant performance and is treated as primary in the remainder of this paper.

Complementary boundary and lesion-wise metrics (HD95, sensitivity, precision, lesion-wise detection) and the full training/validation curves are deferred to the camera-ready version for the reasons given in Section 3.8 and Section 3.11; the subject-level bootstrap intervals reported there provide the available robustness quantification.

### 4.2. Ablation Studies

Table 2 reports the step-by-step ablation. All values are test positive-only Dice from single-seed runs except for the final GDNet row, which reports the mean ± standard deviation over the two-seed and multi-seed evaluation of Section 4.1.

Several findings emerge. First, the 3-slice 2.5D U-Net (A5) outperforms the otherwise-identical 2D baselines (A1, A2) on WT (+0.76 points), TC (+0.63 points), and crucially ET (+0.59 points). This confirms our hypothesis that adding limited inter-slice context to the input materially benefits the smallest, most fragmented tumor sub-region. Second, the 5-slice 2.5D variant (A6) achieves a higher single-run mean foreground Dice (0.8226) than the 3-slice variant (0.8109), but it does so by sacrificing ET (0.6559 versus 0.6694). The wider 5-slice receptive field improves global foreground localization at the cost of fine-grained ET boundary precision, a trade-off we attribute to the dilution of single-slice ET features when averaged over five channels. We therefore retain the 3-slice configuration as the final GDNet because TC and especially ET are clinically the most consequential.

Third, and importantly, the GDNet-style architectural integration (A3) and its region-aware-loss extension (A4) do not outperform the simpler 2.5D U-Net (A5) on positive-only WT/TC/ET. A3 attains a higher mean foreground Dice than A5 (0.8164 versus 0.8109) but loses on every individual region: WT (−1.52), TC (−2.24), ET (−3.39). A4, which adds a region-aware loss intended specifically to help TC and ET, is the worst single configuration tested. This is a notable negative result, which we interpret cautiously given the single split and compute budget: under our modest compute budget, additional architectural complexity is not only unnecessary, it is mildly harmful for the clinically critical sub-regions. We discuss the likely reasons in Section 5.2.

Fourth, light connected-component post-processing (A7) leaves the positive-only Dice essentially unchanged relative to A5, small improvements on a subset of regions are offset by small degradations on others, and the net effect is negligible. By contrast, post-processing did meaningfully improve the all-slice Dice, indicating that connected-component cleanup primarily removes scattered false-positive predictions on otherwise-empty slices. This is consistent with the intuition that post-processing is most effective at the empty-slice/sparse-prediction interface and does not help on dense tumor structures.

Finally, the multi-seed evaluation (Final row) confirms that the chosen configuration is stable: the standard deviation over two seeds is ≤0.0036 on every region, and on WT it is essentially zero (0.0001). This very low variance gives confidence that the gains over A1–A4 are not seed-driven.

### 4.3. Qualitative Analysis

To complement the aggregate metrics, we automatically selected the best, random, and worst test-set predictions ranked by lesion-wise Dice; representative examples are shown in Figure 3, Figure 4, and Figure 5, respectively. Three observations emerge.

Best-case predictions (Figure 3; per-slice Dice ≥ 0.995) are dominated by well-circumscribed, homogeneous tumors with clear contrast on FLAIR (e.g., BraTS-GLI-02083-102 at z = 124, BraTS-GLI-00033-101 at z = 57). GDNet reproduces the lesion contour with sub-voxel accuracy and faithfully recovers both edema (ED) and necrotic-core (NETC) components.

Random-case predictions (Figure 4; per-slice Dice spanning approximately 0.63–0.95) capture the operating regime of GDNet on the bulk of test scans. Here, the model correctly identifies the main tumor mass and the majority of its sub-regions but tends to slightly under-segment thin peritumoral edema rims and small disconnected enhancing foci (e.g., BraTS-GLI-02867-100 at z = 108, BraTS-GLI-02111-103 at z = 86). We occasionally observe small false-positive ED predictions near anatomically heterogeneous regions such as basal-ganglia hyperintensities (e.g., BraTS-GLI-00533-100 at z = 110).

Worst-case predictions (Figure 5; per-slice Dice < 0.15) are heavily concentrated on a small number of anatomically complex scans. Strikingly, five of the seven worst predictions in our test set originate from a single subject (BraTS-GLI-00533-101), in which the tumor is diffusely infiltrative, irregularly shaped, and occupies a large portion of one hemisphere with substantial mass effect. On these slices, GDNet predicts the broad spatial extent of the tumor but misclassifies the internal sub-region labels, producing predominantly ED-labeled voxels where the ground truth contains a structured mix of NETC, ET, and ED. A further failure case (BraTS-GLI-02567-100 at z = 88) involves a small, atypically textured central lesion that the model over-predicts in spatial extent. These observations confirm that the residual catastrophic errors of GDNet are not uniformly distributed across the cohort but rather concentrated on a small minority of anatomically atypical or diffuse cases, a finding with direct implications for clinical deployment and for future hard-case mining.

Each row depicts one axial slice from one subject. Columns, left to right: (a) FLAIR image; (b) expert ground-truth segmentation; (c) GDNet prediction (EMA-averaged 3-slice 2.5D model, seed 43); (d) FLAIR with color-coded prediction overlay. Color code throughout Figure 3, Figure 4 and Figure 5: green, peritumoral edema (ED, label 2); gray, necrotic and non-enhancing tumor core (NETC, labels 1 and 3); red, enhancing tumor (ET, label 4). Cases were selected automatically by ranking all slice-level predictions on the test set by lesion-wise Dice. Examples include BraTS-GLI-02083-102 (z = 124) and BraTS-GLI-00033-101 (z = 57). Best-case slices are dominated by well-circumscribed, homogeneous tumors with high FLAIR–contrast, on which GDNet reproduces the lesion contour with essentially sub-voxel accuracy and faithfully recovers both edema and necrotic-core components.

Each row depicts one axial slice from one subject; column layout and color code as in Figure 3. Cases were selected by uniform random sampling from the full test set, conditioned only on the slice-containing tumor. Examples include BraTS-GLI-02867-100 (z = 108), BraTS-GLI-02111-103 (z = 86), and BraTS-GLI-00533-100 (z = 110). On these scans GDNet correctly identifies the main tumor mass and the majority of its sub-region structure, with the residual error concentrated in (i) mild under-segmentation of thin peritumoral edema rims, (ii) under-prediction of small disconnected enhancing foci, and (iii) occasional false-positive edema predictions near anatomically heterogeneous regions such as basal-ganglia hyperintensities.

Each row depicts one axial slice from one subject; the column layout and color code are the same as in Figure 3. Five of the seven worst predictions in the test set originate from a single anatomically complex subject, BraTS-GLI-00533-101, in which the tumor is diffusely infiltrative, irregularly shaped, and occupies a large portion of one hemisphere with substantial mass effect: on these slices the model correctly captures the broad spatial extent of the lesion but misclassifies the internal sub-region composition, predicting predominantly edema-labeled voxels where the ground truth contains a structured mix of NETC, ET, and ED. A further failure case, BraTS-GLI-02567-100 (z = 88), shows a small, atypically textured central lesion that is over-predicted in spatial extent. The strong clustering of catastrophic errors on a small minority of cases, rather than uniform degradation across the cohort, motivates the future-work directions on hard-case mining and per-case uncertainty estimation discussed in Section 5.5.

## 5. Discussion

### 5.1. Principal Findings

GDNet delivers practical multi-class adult-glioma segmentation on BraTS 2024—competitive among single-GPU, single-model methods—while remaining trainable on a single 8 GB consumer-class GPU. Three findings stand out. First, the combination of EMA weight averaging, mixed tumor-aware sampling, and a 3-slice 2.5D context window produces robust and reproducible test-set performance, with positive-only Dice of 0.791 (WT), 0.736 (TC), and 0.654 (ET) and inter-seed standard deviation ≤ 0.01 Dice points in every region. Second, the all-slice and positive-only Dice scores diverge substantially, by up to ≈ 0.27 Dice points on ET, indicating that the conventional all-slice reporting style can overstate clinically relevant performance, and motivating positive-only Dice as a complementary, stricter metric reported alongside it. Third, a GDNet-style architectural integration with a region-aware loss did not improve positive-only WT/TC/ET Dice over the simpler 2.5D U-Net, suggesting that training strategy and input representation are more influential under modest compute budgets than additional architectural depth.

### 5.2. Why Does the Simpler 2.5D Framework Win?

The negative result on the GDNet-style architectural integration (A3, A4 in Table 2) deserves explicit interpretation. We hypothesize two contributing mechanisms. First, the BraTS 2024 cohort, while sizable by clinical standards, is still modest by deep-learning standards; deeper, more parameter-heavy variants are more prone to overfitting on small tumor sub-regions such as ET that occupy fewer than 1% of intracranial voxels. The compact 2.5D U-Net acts as an inductive-bias-rich regularizer: its small parameter count (≈1.93 M) and its restricted receptive field appear to be a better match for the sub-region statistics than the heavier alternatives we tested. Second, the region-aware loss extension (A4) explicitly increases the weight of TC and ET in the training signal, but it also increases the variance of the gradient on those sub-regions because the WT/TC/ET regional Dice scores are themselves noisy on a small batch. Under EMA stabilization, this extra variance partially offsets the gain from the regional re-weighting and the net effect is slightly negative on TC and ET, the regions the extension was designed to help.

This finding mirrors the broader observation by Isensee et al. [12] that nnU-Net consistently outperforms more specialized pipelines, and supports the position that, for medical image segmentation under modest compute, training-strategy choices dominate architectural innovations.

### 5.3. Why Does 3-Slice Context Beat 5-Slice Context on ET?

The 5-slice 2.5D model (A6) attains a higher single-run mean foreground Dice than the 3-slice model (A5), 0.8226 versus 0.8109, but at the cost of ET, on which it loses 1.35 Dice points (0.6559 versus 0.6694). We interpret this asymmetry as follows. ET is intrinsically a thin, often slice-localized feature; in many tumors it occupies only one or two contiguous axial slices. A 5-slice input window therefore convolutionally mixes the ET signal of the central slice with non-ET surround on the four neighboring slices, diluting the discriminative gradient available to the ET output channel. By contrast, WT and TC are by definition larger structures with stronger inter-slice continuity, and they benefit from the wider context. A 3-slice window preserves enough adjacent context to help WT and TC without diluting the ET signal, and therefore yields a more clinically attractive trade-off. This is consistent with prior 2.5D studies that have found a non-monotonic relationship between context width and small-structure accuracy [17,18].

### 5.4. Relation to the State of the Art

The top entries on the BraTS 2023 and BraTS 2024 leaderboards report Dice values in the 0.85–0.92 range for WT, 0.83–0.89 for TC, and 0.80–0.85 for ET on the official validation cohorts [12,25]. These scores, however, are typically obtained with multi-model ensembles, full 3D nnU-Net or Swin UNETR backbones, test-time augmentation, GAN-based synthetic data augmentation, and the larger 24–64 GB GPUs of the challenge environment. The official BraTS Dice convention also follows the all-slice rule, which (as we have shown in Section 4.1) inflates the score relative to positive-only Dice by between 0.10 and 0.27 Dice points depending on the region. Converted to the all-slice convention, our test scores are 0.890 (WT), 0.923 (TC), and 0.919 (ET), broadly comparable with mid-table BraTS entries under this convention, though we stress this is an indirect comparison across different splits, protocols, and validation-versus-test settings and does not establish parity with high-end 3D models, obtained, however, with a single-GPU, single-model, 1.93 M-parameter system. We view this as encouraging evidence that the framework-level choices in GDNet recover a substantial fraction of the volumetric ceiling while remaining deployable on hardware orders of magnitude more accessible than that of the leaderboard-topping entries. To be precise about what we claim: by “competitive” we mean competitive among single-model, single-GPU methods operating under an 8 GB budget, not parity with leaderboard-topping 3D ensembles. The enhancing-tumor gap is the clearest example: our positive-only ET of 0.654 (all-slice 0.919) remains below the 0.80–0.85 ET range of top 3D systems, and ET is clinically the most consequential sub-region, so this gap is a genuine and acknowledged limitation. Because our numbers and the leaderboard numbers arise from different splits, evaluation protocols, and validation-versus-test settings, the comparison above is indirect; a direct, same-split head-to-head against 3D nnU-Net and Swin UNETR (deferred to future multi-GPU work, Section 5.7) would be required to substantiate any parity claim, and we make none.

### 5.5. Failure Modes and Clinical Implications

The qualitative failure-mode audit (Figure 5) reveals that the residual catastrophic errors of GDNet are highly non-uniform across the cohort: a small number of diffuse, infiltrative, large-mass-effect tumors account for almost all per-slice Dice values below 0.15. For clinical deployment, this has two practical implications. First, an aggregate Dice metric alone is insufficient for risk-stratifying patients: per-case uncertainty quantification, for example through deep-ensemble disagreement or Monte-Carlo dropout, should accompany every segmentation. Second, these atypical cases form a natural target for hard-case oversampling, active learning, and radiologist-in-the-loop refinement. A practical clinical workflow could route low-confidence predictions to expert review while automating the high-confidence majority. Characterizing these hard cases more precisely, they share three features: (a) diffusely infiltrative margins without a sharp tumor–edema boundary, which blurs the WT contour; (b) large mass effect with midline shift that distorts the spatial priors learned from more typical anatomy; and (c) atypical internal composition in which NETC, ET, and ED are finely interdigitated, which the model collapses toward a dominant ED label. Concrete strategies to address them include hard-case oversampling and curriculum or focal re-weighting toward low-Dice subjects, boundary-aware losses (e.g., Hausdorff or boundary-Dice terms) to sharpen infiltrative margins, multi-planar (tri-planar) 2.5D fusion to disambiguate sub-region composition, ET-targeted augmentation to enrich the rare enhancing morphology, and an uncertainty-gated workflow that routes high-disagreement cases to expert review.

### 5.6. Limitations

Our study has several limitations that we declare for transparency. First, we used two independent seeds (43, 44) for the final robustness evaluation; while the observed inter-seed variance is very small, a larger seed sweep (e.g., five seeds) would provide stronger statistical confidence and is planned. Second, we evaluated GDNet on a single cohort (BraTS 2024 adult glioma) with a single subject-level split; external generalization to BraTS-Africa [39], BraTS-PEDs [40], or post-treatment BraTS-GLI cohorts [9] remains to be quantified; accordingly, all quantitative results reported here are specific to the BraTS 2024 cohort and this single split, and our robustness and deployability statements should be read as dataset-specific until external, multi-center validation is completed. Third, our 2.5D representation is axial-only; tri-planar 2.5D fusion combining axial, coronal, and sagittal predictions has been shown to add complementary information [18] and was not explored here. Fourth, GDNet was optimized for Dice; calibration, uncertainty, lesion-wise detection, Hausdorff distance, and clinical-acceptability analyses were not performed. Fifth, our post-processing was restricted to a light connected-component cleanup; more anatomically informed post-processing (for example, brain-mask-constrained morphological operations or atlas-based priors) was not investigated. Sixth, we did not perform direct head-to-head comparison with full 3D nnU-Net or Swin UNETR on the same fixed split; such a comparison is non-trivial within a single 8 GB-GPU budget and is deferred to future multi-GPU work.

### 5.7. Future Work

We see five priority directions: (i) integration of per-voxel uncertainty estimation via deep ensembles or Monte-Carlo dropout, with explicit propagation of uncertainty into the failure-case routing pipeline of Section 5.5; (ii) tri-planar 2.5D fusion combining axial, coronal, and sagittal predictions [18]; (iii) generative augmentation in the style of Kiani Kalejahi et al. [26,27] and Ferreira et al. [25] to enrich training on rare tumor morphologies, particularly the diffuse-infiltrative subtype identified as the dominant residual failure mode; (iv) a controlled head-to-head comparison with a single-GPU 3D nnU-Net baseline and a Swin UNETR baseline under identical compute budgets; and (v) prospective external multi-center validation with explicit reporting of subgroup performance by tumor morphology and by scanner manufacturer.

## 6. Conclusions

We introduced GDNet, a 2.5D multimodal MRI segmentation framework for adult glioma evaluated on the BraTS 2024 cohort. GDNet pairs a compact 2.5D U-shaped encoder–decoder with EMA weight averaging, mixed tumor-aware slice sampling, a compound Cross-Entropy + Soft-Dice loss, AdamW with warm-up plus cosine annealing, and Automatic Mixed Precision. On the BraTS 2024 test partition, GDNet achieves test positive-only Dice scores of 0.791 ± 0.000 for whole tumor, 0.736 ± 0.003 for tumor core, and 0.654 ± 0.004 for enhancing tumor across two independent training seeds, with an inter-seed standard deviation ≤ 0.01 Dice points on every region. A systematic ablation shows that EMA + mixed tumor sampling and the 2.5D context window are the dominant sources of improvement and reveals a robust negative result: a GDNet-style architectural integration with a region-aware loss does not improve positive-only WT/TC/ET Dice over the simpler 2.5D U-Net. A failure-mode audit identifies a small number of diffuse, infiltrative tumors as the dominant source of residual catastrophic error, motivating future work on uncertainty estimation and hard-case oversampling. GDNet offers an accuracy-to-compute trade-off that is favorable for clinical deployment in settings without access to high-end volumetric GPU hardware and supports the broader thesis that under modest compute budgets, training strategy and input representation are more influential than additional architectural depth.

## Figures and Tables

**Table 1:** Final 3-slice 2.5D GDNet performance on BraTS 2024 across two seeds (43, 44).

Partition	Mean Foreground	WT	TC	ET
Validation (positive-only)	0.8326 ± 0.0006	0.8051 ± 0.0022	0.7570 ± 0.0040	0.6831 ± 0.0094
Test (positive-only)	0.8198 ± 0.0002	0.7910 ± 0.0001	0.7359 ± 0.0035	0.6536 ± 0.0036
Validation (all-slice)	0.9297 ± 0.0011	0.8962 ± 0.0028	0.9233 ± 0.0006	0.9208 ± 0.0007
Test (all-slice)	0.9272 ± 0.0004	0.8901 ± 0.0026	0.9234 ± 0.0011	0.9189 ± 0.0011

**Table 2:** Ablation: test positive-only Dice across the experimental pipeline.

Step	Configuration	Mean FG	WT	TC	ET
A1 (7.1d)	2D U-Net + EMA + mixed sampling	0.8058	0.7902	0.7396	0.6635
A2 (7.3b)	A1 + tumor-centered crop + LR reset	0.8066	0.7836	0.7338	0.6538
A3 (8)	GDNet-style architectural integration (2D)	0.8164	0.7826	0.7235	0.6355
A4 (8.1)	A3 + region-aware (WT/TC/ET) loss	0.8150	0.7752	0.7135	0.6243
A5 (9)	3-slice 2.5D U-Net (final GDNet)	0.8109	0.7978	0.7459	0.6694
A6 (9.1)	5-slice 2.5D U-Net	0.8226	0.7961	0.7430	0.6559
A7 (9.2)	A5 + light post-processing	0.8140	0.7973	0.7453	0.6684
Final	3-slice 2.5D GDNet, 2-seed mean ± std	0.8198 ± 0.0002	0.7910 ± 0.0001	0.7359 ± 0.0035	0.6536 ± 0.0036

## Data Availability

The BraTS 2024 multi-parametric MRI dataset analyzed in this study is publicly available through the BraTS challenge organizing committee at https://www.synapse.org/brats (accessed on 23 November 2025), subject to the corresponding Data Use Agreement.
